# Adult minimal-change disease: observational data from a UK centre on patient characteristics, therapies, and outcomes

**DOI:** 10.1186/s12882-018-0999-x

**Published:** 2018-08-16

**Authors:** Anthony Fenton, Stuart W. Smith, Peter Hewins

**Affiliations:** Department of Renal Medicine, Elizabeth Hospital Birmingham, University Hospitals Birmingham NHS Foundation Trust, Birmingham, UK

**Keywords:** Minimal change disease, Outcomes, Patient characteristics, Treatments

## Abstract

**Background:**

Minimal change disease (MCD) is a common cause of the nephrotic syndrome in adults with limited evidence on its treatment and prognosis. We examined the presenting characteristics, treatments, and outcomes of adult patients with MCD in our centre.

**Methods:**

This was an observational cohort study using retrospectively-collected data. All patients who had a renal biopsy reported as MCD between 1996 and 2012 were included, and data were collected at baseline and during follow-up. Statistical analysis included Cox-regression analysis to examine which factors were associated with risk of relapse.

**Results:**

Seventy-eight patients were included, and had a median age of 36 years, and were 60% male and 73% white. Median follow-up time was 72 months. 37% were in AKI at presentation, which was significantly associated with a lower serum albumin and older age. Although 10% were steroid-resistant, 98% achieved remission at a median time of 5 weeks. 61% relapsed, at a median time of 11 months, and patients had a median number of 2 relapses during follow-up. A higher eGFR was associated with an increased risk of relapse (hazard ratio 1.18 [1.03–1.36] per 10 mL/min increase in eGFR), and females were significantly more likely than males to have an early relapse. Nearly half of the cohort required an additional immunosuppressive agent on top of glucocorticoids, the most commonly used being calcineurin inhibitors. Five patients subsequently developed FSGS: these patients had a lower baseline creatinine, a higher serum albumin, a longer time to remission, and were more likely to be steroid-resistant. Follow-up renal function was generally preserved, but follow-up creatinine was higher in those who had presented with AKI, and in those who had been commenced on a RAS inhibitor after biopsy. Infection requiring admission, diabetes mellitus and venous thromboembolism developed in 14%, 12%, and 12% of patients respectively.

**Conclusions:**

Nearly all adults with MCD achieve remission, but relapses and disease- and therapy-related complications are common. In our cohort, eGFR and gender were associated with risk of relapse, and these previously undescribed associations could be explored further in future work.

## Background

Minimal change disease (MCD) refers to the occurrence of the nephrotic syndrome (NS) with a renal biopsy showing normal glomeruli on light microscopy, no staining on immunofluorescence microscopy, and diffuse foot process effacement on electron microscopy [1]. It accounts for approximately 11–16% of NS in adults and is the third commonest cause after membranous nephropathy and focal segmental glomerulosclerosis (FSGS) [1–4].

Historically, untreated MCD was associated with a slow rate of spontaneous remission, and with life-threatening complications of NS including sepsis and thrombosis. MCD is therefore usually treated with immunosuppression, the cornerstone being glucocorticoids. A 2008 Cochrane review identified only three randomised controlled trials (RCT) examining the first-line treatment of MCD in adults, and these had small numbers of participants and inadequate power to detect differences in therapeutic efficacy [5]. The review was also unable to identify any RCT data for the treatment of relapsing or steroid-resistant disease [5]. Guidelines for the treatment of adult MCD are therefore based largely on extrapolation from RCTs in children and observational studies.

Although there are several published case series of patients with adult MCD, there are relatively few studies on the treatment and outcomes of adults with MCD [6–13]. We collected data on the baseline characteristics, treatments, and outcomes of adult patients with MCD in our UK renal centre.

## Methods

This was an observational cohort study using retrospectively-collected data. The cohort comprised adults in our renal centre with MCD, who were identified by a search of our pathology database for all renal biopsies performed between 1996 and 2012 which were reported as MCD. Patients with multiple biopsies were included in the dataset only once. Demographic, clinical, and laboratory data were collected from the hospital electronic database and clinic letters, and baseline laboratory data were the closest available to the time of renal biopsy within seven days. Outcome data were collected up to December 2013, and patients lost to follow-up were censored at the point of their last clinic visit.

Estimated glomerular filtration rate (eGFR) was calculated using the four-variable Modification of Diet in Renal Disease (MDRD) study equation [14]. Proteinuria was measured using a urine albumin:creatinine ratio (uACR) or a 24-h urine collection, and sometimes during follow-up a urine dipstick for the determination of remission or relapse. Haematuria was defined as ≥45 red blood cells/μL on flow cytometry of a mid-stream urine specimen (the upper limit of the reference range in our centre). Acute kidney injury (AKI) was defined as a rise in serum creatinine of ≥50% known or presumed to have occurred in the preceding seven days, and episodes of AKI were staged 1–3 by serum creatinine as per the Kidney Disease Improving Global Outcomes (KDIGO) AKI guideline [15].

Complete remission (CR) was defined as a uACR < 30 mg/mmol or a urine dipstick negative for protein. In patients who attained CR, a relapse was defined as a uACR > 220 mg/mmol or an increase in proteinuria sufficient for the managing clinician to alter medical therapy. Steroid-resistance was defined as persistent proteinuria despite high-dose prednisolone sufficient for the managing clinician to introduce an additional immunosuppressive agent. Steroid-dependence was defined as a relapse while the patient was on tapering steroid therapy, or within two weeks of completing steroid therapy. Frequently relapsing disease was defined as three or more relapses in a year.

Statistical analyses were performed using IBM SPSS Statistics for Macintosh, Version 22 (Armonk, NY: IBM Corp, 2013). Complete case analysis was used. Categorical variables are expressed as a number and percentage, and continuous variables are expressed as a mean and standard deviation (SD) or median and interquartile range (IQR). Differences between continuous variables were tested for statistical significance using Student’s *t*-test or, for non-parametric analyses, the Mann-Whitney U test or Kruskal-Wallis test depending on the number of groups being compared. Due to sample size considerations, exact methods were used for the analysis of categorical variables (Fisher’s exact test or an exact chi-square test computed using the Monte Carlo method). Kaplan-Meier plots were generated to show time to CR and time to relapse, and Cox regression analysis was used to assess the associations between baseline characteristics and time to CR and time to relapse. Statistical significance was taken to be a *P* value < 0.05.>

## Results

Seventy-eight patients had a renal biopsy consistent with MCD between 1996 and 2012 and were followed up for a median time of 72 months (range 6–190). Demographic, clinical, and laboratory data from the time of renal biopsy are shown in Table 1. Six (8%) patients were suspected of having secondary MCD, with the underlying cause being chronic lymphocytic leukaemia in one patient, Kimura disease in one patient, and drug-related in four patients (three due to non-steroidal anti-inflammatory drugs, and one due to either tiopronin or captopril). Twenty-one (27%) patients had a history of NS in childhood.

**Table 1:** Baseline characteristics of the study population

Characteristic	Whole cohort (*N* = 78)	Subgroup: first presentation (*N* = 52)	Completeness of data (%)
Age (years)	36 (25–50)	47 (31–63)	100
Gender (male)	47 (60)	30 (58)	100
Ethnicity			99
White	56 (73)	40 (77)	
South Asian	14 (18)	6 (11)	
Black	6 (8)	5 (10)	
Other	1 (1)	1 (2)	
Serum creatinine (μmol/L)^a^	91 (70–122)	105 (78–138)	100
Estimated GFR (mL/min/1.73m^2^)^a^	82 (57–106)	68 (46–92)	100
Acute kidney injury	22 (28)	19 (37)	100
Proteinuria			82
ACR (mg/mmol) (*N* = 51)	594 (458–899)	609 (505–913)	
24 h collection (g/24 h) (*N* = 13)	5.0 (4.0–8.3)	4.5 (3.4–8.2)	
Serum albumin (g/L)	22 (17–30)	21 (17–24)	97
Serum cholesterol (mmol/L)	9.0 (7.3–12.1)	9.0 (7.4–12.4)	83
Haematuria	9 (15)	4 (10)	80

Baseline characteristics of the whole study population and of the subgroup of patients biopsied during their first presentation with nephrotic syndrome. Continuous variables are expressed as a median (interquartile range) and categorical variables as a number (percentage). *MCD* minimal change disease; *GFR* glomerular filtration rate, estimated by 4-variable MDRD equation; *ACR* albumin-to-creatinine ratio. ^a^Includes patients with acute kidney injury

The cohort included 17 patients who already had a diagnosis of MCD, and were biopsied either in the context of relapsing disease or to check for evidence of calcineurin inhibitor (CNI) toxicity. The other 61 patients were all presenting for the first time, presumably nephrotic, although only 52 patients had available proteinuria data to confirm NS.

### Patients with their first presentation of MCD

The baseline characteristics of the 52 patients presenting for the first time with NS are shown separately in Table 1. At presentation, 19 (37%) patients had AKI (16 of these had imaging specifically of the renal veins, none of whom had evidence of thrombosis). Those with AKI were older (53 vs 40 years, *P* = 0.028), and had a lower serum albumin (19 vs 23 g/L, *P* = 0.032). There was no statistically significant association between having AKI at presentation and level of proteinuria, the presence of haematuria, gender, or ethnicity.

### Initial therapy and response

Of the 52 patients who were presenting with NS for the first time, one entered CR spontaneously before taking any immunosuppression. The other 51 patients (including the six with suspected secondary MCD) all received daily oral prednisolone: most patients (36 [72%]) received 60 mg (range 30–60, median 60). Initial tapering prednisolone therapy was continued for a median total duration of 6 (IQR 4–12) months.

Forty-six (90%) patients were steroid-responsive, i.e. attained CR with prednisolone only. The other five (10%) patients were judged steroid-resistant by the treating clinician and commenced an additional immunosuppressive agent after 3 weeks in two patients, 14 weeks in one patient, and 17 weeks in two patients. Three patients received a CNI (two achieved CR, at 1 week and 26 weeks; the third patient never achieved CR and underwent a repeat biopsy which showed FSGS), and two received cyclophosphamide (both achieved CR, at 4 weeks and 14 weeks).

**Fig. 1:**
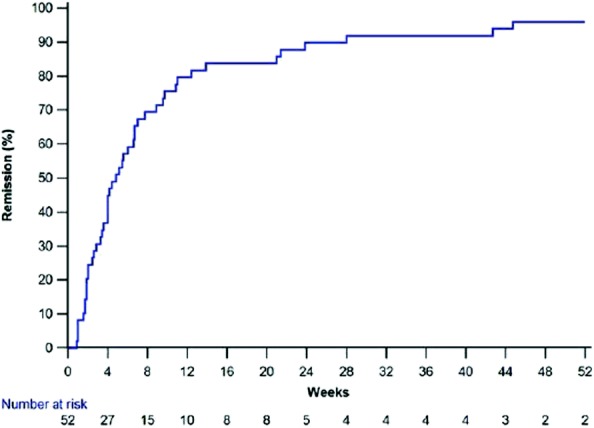
Kaplan-Meier plot showing the cumulative proportion achieving complete remission from the time of commencing initial therapy

Overall, 51 (98%) of 52 patients attained CR. Figure 1 shows the cumulative proportion of the cohort who achieved CR from the time of commencing initial therapy. Median time to CR was 5 (IQR 2–11) weeks, and the number (percentage) who attained CR were 22 (42%) by 4 weeks, 34 (65%) by 8 weeks, 39 (75%) by 12 weeks, and 41 (79%) by 16 weeks.

The associations between baseline characteristics and time to CR are shown in Table 2. There were no significant associations between any of the examined baseline variables and time to CR. Consistent with this, early responders (CR within 6 weeks, 29 patients) and late responders (CR after 6 weeks, 19 patients), had no significant differences with regards to age, gender, ethnicity, serum creatinine, AKI at presentation, serum albumin, level of proteinuria, serum cholesterol, or presence of haematuria.

**Table 2:** Associations between patient characteristics and time to remission and relapse by Cox-regression analysis

Characteristic	Remission		Relapse	
	HR (95% CI)	*P*	HR (95% CI)	*P*
Age (years)^a^	0.97 (0.83–1.12)	0.64	0.81 (0.64–1.01)	0.06
Gender		0.62		0.32
Male	1		1	
Female	0.86 (0.49–1.54)		1.46 (0.70–3.03)	
Ethnicity		0.75		0.93
White	1		1	
South Asian	0.60 (0.21–1.74)		1.01(0.30–3.38)	
Black	0.72 (0.27–1.91)		0.61 (0.14–2.57)	
Other	0.72 (0.10–5.32)		N/A	
Serum creatinine (μmol/L)^b^	0.99 (0.95–1.03)	0.72	0.97 (0.90–1.03)	0.30
Estimated GFR (mL/min/1.73m^2^)^a^	1.00 (0.91–1.09)	0.98	1.18 (1.03–1.36)	0.016
Baseline AKI	1.12 (0.61–2.05)	0.71	0.71 (0.33–1.53)	0.38
Proteinuria				
ACR (mg/mmol)^c^	1.04 (0.93–1.17)	0.51	1.06 (0.94–1.20)	0.34
24 h collection (g/24 h)^d^	1.25 (0.99–1.59)	0.06	1.01 (0.84–1.21)	0.92
Serum albumin (g/L)^e^	0.88 (0.69–1.13)	0.32	1.10 (0.83–1.46)	0.50
Serum cholesterol (mmol/L)^d^	1.02 (0.95–1.11)	0.53	1.02 (0.92–1.14)	0.65
Haematuria	0.79 (0.24–2.62)	0.70	0.33 (0.04–2.47)	0.28
Steroid resistance			1.45 (0.44–4.80)	0.54
Time to remission (weeks)^d^			0.99 (0.96–1.02)	0.68
Duration of initial course of prednisolone^f^			0.88 (0.72–1.06)	0.18

*HR* hazard ratio; *CI* confidence interval; *GFR* glomerular filtration rate; *AKI* acute kidney injury; *ACR* albumin-to-creatinine ratio. ^a^per 10 units; ^b^per 20 units; ^c^per 100 units; ^d^per 1 unit; ^e^per 5 units; ^f^per 1 month of therapy, analysed after excluding those who relapsed while still on their initial prednisolone

### Relapses

**Fig. 2:**
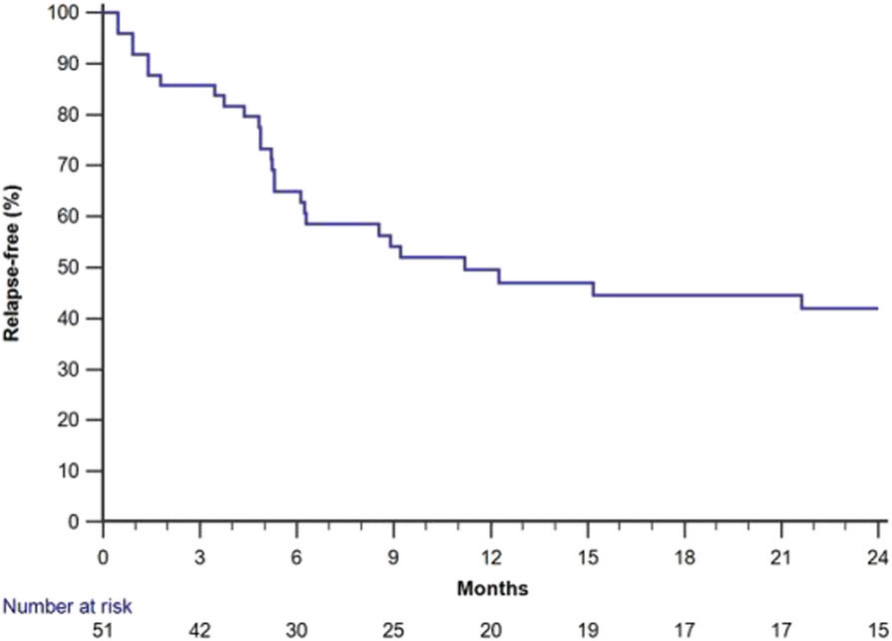
Kaplan-Meier plot showing the cumulative proportion of the cohort who relapsed from the time of attaining complete remission

Thirty-one patients (61% of those patients who had achieved CR) relapsed, including 22 (43%) patients who had multiple relapses. Figure 2 shows the cumulative proportion of the cohort who had relapsed from the time of attaining CR. By six months, 17 (33%) patients had relapsed, including 11 who were steroid-dependent, and median time to relapse was 11 months. Throughout follow-up, patients had a median of two relapses (IQR 0–4, range 0–11), and ten (13%) patients had frequently relapsing disease.

The single patient who had spontaneously entered CR never relapsed. Of the 46 steroid-responsive patients, 28 (61%) relapsed. All 28 patients were put back on high-dose prednisolone and all re-attained remission. Of the 11 steroid-dependent patients, five were treated with a CNI in addition to prednisolone.

Of the patients initially designated steroid-resistant, both patients who achieved CR after cyclophosphamide treatment went on to relapse: one was given a CNI in addition to high-dose prednisolone and attained remission, and the other was given high-dose prednisolone but died before remission was achieved (cause of death unavailable). Of the two patients who achieved CR after a CNI, one relapsed but re-entered remission with high-dose prednisolone.

The associations between baseline patient characteristics and risk of relapse are presented in Table 2. A higher eGFR was associated with a significantly increased risk of relapse (HR 1.18 [1.03–1.36] per 10 mL/min increase in eGFR, *P* = 0.016). This association between baseline kidney function and relapse was borne out when relapsers were compared to non-relapsers; relapsers had a lower baseline creatinine (93 vs 119 μmol/L, *P* = 0.019) and higher eGFR (85 vs 57 mL/min, *P* = 0.015).

To analyse the association between baseline characteristics and time to relapse further, early relapsers (< 6 months, 17 patients), late relapsers (> 6 months, 12 patients), and non-relapsers (20 patients) were compared. Females were significantly more likely than males to experience an early relapse (57% vs 18%, *P* = 0.006). There was no significant association with age, ethnicity, eGFR, serum albumin, level of proteinuria, the presence of haematuria, AKI at presentation, steroid-resistance, time to CR, or duration of initial prednisolone therapy.

### Therapeutic agents

Seventy-six (97%) patients were treated with prednisolone at some point during follow-up. Patients received a median of two courses (IQR 1–3, range 0–7), and had a median total prednisolone exposure of 76 (IQR 32–168) weeks during follow-up.

The proportion of the whole cohort (*N* = 78) treated with second-line immunosuppressants are shown in Table 3. Thirty-three (42%) patients required at least one additional agent at some point during follow-up. Calcineurin inhibitors were the most common additional agent used, being used in 25 (32%) patients (15 received ciclosporin and 10 received tacrolimus), in whom the median total duration of CNI exposure was 35 (IQR 16–45) months. They were used in three patients for steroid-resistance (two of whom achieved CR), and in 22 for relapsing disease (18 of whom achieved remission, although 15 of these went on to relapse again).

**Table 3:** Use of second-line immunosuppressants

Immunosuppressant	*N* (%) of cohort treated	Duration of therapy	Achieved CR (*N*)	Relapsed (*N*)	Time to relapse (months)
Calcineurin inhibitors	25 (32)	18 (10–34)^a^	21	16	7 (2–22)
Cyclophosphamide	10 (13)	9 (8–10)^b^	9	6	20 (5–44)
Levamisole	7 (9)	7 (4–38)^b^	6	5	10 (3–14)
Mycophenolate mofetil	6 (8)	10 (3–29)^a^	4	3	47 (25–56)
Rituximab	4 (5)	N/A	4	0	N/A

The table shows the number (percentage) of the total cohort treated with each class of immunosuppressant, with the median (IQR) duration of the course, and the number who achieved complete remission. The number who went on to relapse is shown, with the median (IQR) time to relapse. Cyclophosphamide was given orally in all cases. If a patient received more than one course of a particular class of agent, the median therapy duration of therapy and the remission/relapse rates relate to the first course given. ^a^months; ^b^weeks

Oral cyclophosphamide was used in 10 (13%) patients, for a median duration of 10 (IQR 7–13) weeks. It had been used in two patients for steroid-resistance (both achieved remission but went on to relapse), and in eight for relapsing disease (seven achieved CR, four of whom relapsed again). Rituximab was used in four patients for relapsing disease, all of whom achieved CR, and none went on to relapse during follow-up. Other agents used for relapsing disease included levamisole in seven (9%) and mycophenolate mofetil in six (8%) patients. Forty-nine (63%) patients were treated with a renin-angiotensin system (RAS) inhibitor at some point during follow-up.

### Subsequent renal biopsies and FSGS

Sixteen patients had subsequent renal biopsies, all of whom had received a CNI. Seven of these had evidence of CNI damage, and five showed FSGS.

Those who were subsequently diagnosed with FSGS (*N* = 5) had a significantly lower baseline serum creatinine (58 vs 109 μmol/L, *P* = 0.028) and higher serum albumin (27 vs 20 g/L, *P* = 0.047). Patients with FSGS had a longer time to remission (43 vs 4 weeks, *P* = 0.039) and were more likely to be steroid resistant (60% vs 9%, *P* = 0.014). There were no significant differences in those who subsequently developed FSGS with regards to age, gender, ethnicity, level of proteinuria, the presence of haematuria, or AKI at presentation.

### Outcomes

At last follow-up, median serum creatinine was 87 (68–108) μmol/L, median eGFR was 78 (59–104) mL/min, and one patient had renal failure requiring haemodialysis (this patient had a relapse which precipitated dialysis-requiring AKI, which failed to recover before death six weeks later). A higher follow-up creatinine was found in those who were in AKI at presentation (91 vs 82 μmol/L, *P* = 0.050), and in those who had been commenced on a RAS inhibitor at some point after their renal biopsy (94 vs 78 μmol/L, *P* = 0.029). There was no significant association between follow-up creatinine and the presence of haematuria, being steroid-resistant, having one or more relapses, or a diagnosis of FSGS.

Twenty-seven (35%) patients had at least one hospital admission during follow-up. Thirty-two (41%) patients had an episode of AKI, either at presentation or during follow-up, including 12 (15%) patients who had an episode of stage 3 AKI. Although more patients treated with a RAS inhibitor developed an episode of AKI compared to those not on a RAS inhibitor, this difference was not statistically significant (24% vs 17%, *P* = 0.57). Other disease and therapy-related complications included 11 (14%) who had an admission-requiring infection, and 9 (12%) who developed diabetes mellitus - of these, one required treatment with insulin, six required oral agents, and two were diet-controlled. Six (8%) patients died during follow-up, at a median age of 84 years (range 79–85).

Nine (12%) patients developed a venous thromboembolism (VTE). Seven of these were nephrotic at the time of VTE, and none were on prophylactic anticoagulation at the time. There was no unit protocol for prophylactic anticoagulation below a specific cut-off of serum albumin in patients with MCD, but six (8%) patients had received prophylactic anticoagulation during episodes of NS, none of whom developed a VTE.

## Discussion

There is a paucity of data available on the presenting features, treatments, and prognosis of MCD in adults, and so we retrospectively collected data to describe a cohort of 78 adults with MCD managed in our renal centre.

On presentation with the NS, AKI is common: 37% of our cohort had AKI at first presentation, a similar proportion to that reported in other studies [6, 13, 16]. These patients were significantly older (it is known that older age is a risk factor for AKI), had more severe hypoalbuminaemia (which may be associated with intravascular depletion), and had a significantly higher serum creatinine at last follow-up, findings all consistent with previous data [9, 12]. Ten per cent of our cohort also had microscopic haematuria at presentation, which has previously been reported to be present in approximately 20% in paediatric and adult cohorts [6, 11, 12, 16, 17].

Ninety per cent of newly diagnosed MCD patients were steroid-responsive, and nearly all patients achieved remission with immunosuppressive therapy. Median time to remission was five weeks, which was similar to that reported in other cohorts [6, 7, 13, 16]. There were no baseline characteristics significantly associated with time to remission. It is notable that our rate of steroid resistance is not higher than that reported in other cohorts. Current KDIGO guidelines for adult MCD propose treating for up to 16 weeks with high-dose glucocorticoid treatment before considering patients to be steroid-resistant but the strength of the evidence for this recommendation is low (level 2C) and it is based on a comparatively small number of adult patients in observational studies. Clinicians at our centre have generally taken the view that extended high-dose steroid treatment presents an unacceptable risk of inducing significant complications and have therefore tended to opt for steroid weaning and introduction of additional immunosuppressant agents at an earlier time point. Accordingly, we employed a pragmatic definition of steroid resistance in our cohort based on clinician decision to change treatment rather than using the KDIGO definition.

Despite the vast majority of patients achieving remission, relapse was common, occurring in 61% of the cohort, with a median time to relapse of 11 months. The risk of relapse was increased in those with a higher baseline eGFR, and there was a significantly increased risk of early relapse in females. These associations have not been found in other MCD cohorts, including a recent study which looked specifically at predictors of relapse in adult MCD [18]. There is evidence from other MCD cohorts that younger age is associated with an increased rate of relapse, but we did not observe this association in our cohort [6–8, 16, 18]. Duration of initial prednisolone treatment and rate of tapering might impact on relapse risk and slow tapering is proposed in KDIGO guidelines on this basis. At our centre, prednisolone treatment duration and tapering were determined by the treating clinician rather than by protocol. Initial prednisolone therapy (in a tapering regimen) was continued for a median of 6 (IQR 4–12) months indicating that most patients were not treated with shorter course steroids but notably we found no significant association between the total duration of initial prednisolone therapy and risk of relapse.

Nearly half of the total cohort received at least one immunosuppressant in addition to prednisolone. Calcineurin inhibitors were the most common agents used: a third of our cohort received one at some point, and the majority achieved remission. In this context, serious infection and new onset diabetes mellitus still developed at comparatively high rates (14% and 12%, respectively). Whilst all immunosuppressant agents can potentially induce significant side-effects, we would argue that it is unlikely that more extended high dose steroid treatment before introducing other agents represents the optimal approach for managing adult MCD.

There is also a growing body of evidence demonstrating the effectiveness of rituximab in adults with relapsing MCD [19–23]; four patients in our cohort were treated with rituximab, and all achieved sustained remission with no relapses during follow-up. Since the time of data collection, more patients with relapsing MCD in our centre have successfully been treated with rituximab which we have reported elsewhere [24]. We believe that further research to determine the utility of rituximab as a means to reduce dependence on high-dose steroid and other immunosuppressant agents in adult MCD would be of considerable value.

Two-thirds of our cohort were commenced on a RAS inhibitor at some point after their diagnosis of MCD, and their use was associated with a significantly higher follow-up serum creatinine. Although RAS inhibitors are often used in proteinuric kidney disease, there is no rationale for the use of RAS inhibitors in cases of MCD where the proteinuria resolves quickly with immunosuppression. RAS inhibitors may increase the risk of AKI which is already common in MCD especially during nephrotic episodes. Although there was no significant difference in the number of patients who developed AKI between those treated with a RAS inhibitor and those not treated, we did not have data on whether patients were on the RAS inhibitors during relapses when patients would be at the highest risk of AKI.

Renal function was well-preserved at the end of follow-up, and only one patient was requiring dialysis at last follow-up, consistent with other published data. Five patients were diagnosed with FSGS, and as expected these patients were more likely to be steroid resistant and had a significantly longer time to CR. Interestingly, neither more severe hypoalbuminaemia nor higher serum creatinine at the time of presentation were indicators of a higher risk of FSGS.

This study is one of the larger cohorts of adult MCD patients published to date, but we recognise that it has limitations. The data were retrospectively collected and were, therefore, dependent upon the accuracy and completeness of hospital databases and clinic letters. The data were neither created nor collected to answer a specific hypothesis, but have been used to provide an overview of the adult MCD cohort in our centre.

## Conclusions

Nearly all adults with MCD achieve remission, but relapses and disease-related complications are common. Several characteristics associated with risk of relapse in our cohort have not been described previously and could be explored further in future work. Patients commonly require extra immunosuppression in addition to the standard treatment of glucocorticoids, and adverse effects are seen in a significant proportion of patients.

There remains a paucity of evidence to guide the management of adult MCD. Large, prospective, observational cohort studies would provide a better understanding of the natural history and prognosis of adults with MCD, and there is a real need for RCTs to guide the treatment of steroid-resistant and frequently relapsing disease, including further investigation of the efficacy of rituximab.

## Abbreviations

AKI: Acute kidney injury
CNI: Calcineurin inhibitor
CR: Complete remission
eGFR: Estimated glomerular filtration rate
FSGS: Focal segmental glomerulosclerosis
IQR: Interquartile range
KDIGO: Kidney Disease: Improving Global Outcomes
MCD: Minimal change disease
MDRD: Modification of diet in renal disease
NS: Nephrotic syndrome
RAS: Renin-angiotensin system
RCT: Randomised controlled trial
SD: Standard deviation
uACR: Urine albumin:creatinine ratio
UK: United Kingdom
VTE: Venous thromboembolism
